# Dr. Trotter, on the Jennerian Inoculation

**Published:** 1801-01

**Authors:** T. Trotter

**Affiliations:** Plymouth Dock


### *To the Editors of the Medical and Phyfical Journal.*

Gentlemen,

It will, I prefume, be gratifying to the numerous lift of phyficians and furgeons, who have patronized Dr. Jenner's difcovery, to hear that the inoculation of the vaccine difeafe is making rapid progrefs in the fleet. This bufinefs has been much facilitated by the kind attention with which the matter is fupplied to our furgeons, as they arrive from fea, by Meffrs. Dunning and Little, two eminent furgeons in this town. I embrace this opportunity, as the executive medical officer of the fleet, to offer them my beft and moft grateful thanks in the name of the public fervice. Mr. Stephen Hammick, of the Royal Hofpital, is alfo intitled to this public acknowledgement for fimilar kindnefs.

If the value of any improvement in the practice of medicine is to be eftimated by its contracting the fcope of human mifery, the Jennerian Inoculation will be defervedly recorded as one of the greateft bleffings to the navy of Great-Britain that was ever extended to it. Since the publication of my fecond volume of Med. Nautica, not lefs than eleven fhips of the line, befide frigates, have imported, from various fources, the variolous contagion.

In the flag fhip of the commander in chief, while refitting at Portfmouth, laft fummer, it extended to twelve cafes before it was checked. Dr. Felix, who then officiated as furgeon of the Ville de Paris, reports to me, that more men were found who were unconfcious of having had the Small Pox, than had been known in any other fhip of the line. One fhip of feventy-four guns, carried it to fea in the fpring, and buried five feamen during the cruize; another has carried it abroad, the fate of which which I have not yet learned. The Impetueux, now in harbour, received the Small-pox from Dock, where they rage with confiderable mortality. A lieutenant in this fhip fet the example of the new inoculation to the feamen; fixteen have taken it, and others, from fuperftitious motives, ftill hold back.

When the candid enquirer, at fome future period, fhall canvafs thefe obvious facts, it will be feen whether I was acting an inconfiderate part in recommending the practice in the navy, when it firft came before the public, as malignantly ftated by fome cold-hearted critic of the prefent day.

---

In the fpring of the year, when fevers of the low kind were rather general in our fhips, the *affufions of cold water*, as recommended by Dr. Currie, of Liverpool, were found of great fervice by Meffrs. Farquhar, of the Captain, and M'Grath, of the Ruffel. Thefe gentlemen had their hands full for a length of time; but I am difpofed to mention the trials here, that the learned author may know that his advice is not thrown away upon us. Other particulars will appear, in due time, in our future Hiftory of Health.

---

Since my laft communication on Coxwell's Concrete Citric Acid, experiments have been directed by the Right Honourable Lords Commiffioners of Admiralty, with their ufual benevolence, and the reports of feven fhips uniformly confirm the firft trials. This preparation of the lemon has now got to the mefs table of all our officers, and will, no doubt, become general. The fubject of fcurvy is thus finally difcuffed, unlefs Sir Francis Milman, Bart. fhould produce fome frefh arguments to fupport his theory, in a new edition of his elegant work. The apothecary will alfo find a pure and permanent folution of this acid a valuable addition to the fhop.

With refpect to my own ftudies, as well as a moft cruel rheumatifm will permit, I proceed as heretofore, like Horace,

Condo et compono quæ mox depromere possim.

I have not heard a word of the Nitrous Fumigation from the furgeons for thefe many months. The mafterly difcipline enforced by the illuftrious officer at the head of the fleet, has effectually fubdued all wavering opinions on that fubject. We fhall, however, always be glad to receive hints from phyficians at the weft end of the town; and let them be affured, there is no prejudice here, but the utmoft defire to improve. I am,

GENTLEMEN,

With much deference,

Your obedient humble fervant,

T. TROTTER.

*Plymouth Dock,*

